# Estimating Nitrogen Load Resulting from Biofuel Mandates

**DOI:** 10.3390/ijerph13050478

**Published:** 2016-05-09

**Authors:** Mohammad Alshawaf, Ellen Douglas, Karen Ricciardi

**Affiliations:** 1 Environmental Technology Management Department, Kuwait University, P.O Box 5969, Safat 13060, Kuwait; 2 School for the Environment, University Of Massachusetts Boston, Boston, MA 02125, USA; ellen.douglas@umb.edu (E.D.); Karen.Ricciardi@umb.edu (K.R.)

**Keywords:** biofuels, ethanol, nitrogen, SPARROW, EISA

## Abstract

The Energy Policy Act of 2005 and the Energy Independence and Security Act (EISA) of 2007 were enacted to reduce the U.S. dependency on foreign oil by increasing the use of biofuels. The increased demand for biofuels from corn and soybeans could result in an increase of nitrogen flux if not managed properly. The objectives of this study are to estimate nitrogen flux from energy crop production and to identify the catchment areas with high nitrogen flux. The results show that biofuel production can result in an increase of nitrogen flux to the northern Gulf of Mexico from 270 to 1742 thousand metric tons. Using all cellulosic (hay) ethanol or biodiesel to meet the 2022 mandate is expected to reduce nitrogen flux; however, it requires approximately 25% more land when compared to other scenarios. Producing ethanol from switchgrass rather than hay results in three-times more nitrogen flux, but requires 43% less land. Using corn ethanol for 2022 mandates is expected to have double the nitrogen flux when compared to the EISA-specified 2022 scenario; however, it will require less land area. Shifting the U.S. energy supply from foreign oil to the Midwest cannot occur without economic and environmental impacts, which could potentially lead to more eutrophication and hypoxia.

## 1. Introduction 

The growing concern over high crude oil prices and the U.S. dependency on foreign oil led the U.S. Congress to pass the Energy Policy Act of 2005 and Energy Independence and Security Act (EISA) of 2007. Both acts provide economic incentives to use renewable fuels, mainly ethanol, to meet the energy demands in the transportation sector and to reduce dependency on foreign oil. The EISA set a mandatory renewable fuel standard requiring that at least 136 billion liters of biofuels be used by 2022 as transportation fuel (Table 1).

The Mississippi River basin drains approximately 40% of the United States and is it the largest contributor of nutrients to the northern Gulf of Mexico [2,3,4]. Nutrients can come from many sources, such as discharge from sewage plants, atmospheric nitrogen deposition, agriculture and urban development. However, excess fertilizers from agricultural fields are the largest source of nutrient runoff to the Mississippi River causing the formation of the hypoxia region in the northern part of the Gulf of Mexico (a region of low concentrations of dissolved oxygen) [5]. The average annual total nitrogen flux to the northern Gulf of Mexico from 2005 to 2009 was 1.26 million metric tons [4]. The rate and the concentration of nitrate losses in drainage tiles vary with soil type, climate conditions, fertilizer application rate and timing, drain spacing, cover crops, crop yield and water table control practices [6]. 

The increased demand for biofuels from feedstock, such as corn and soybeans, could result in an increase of nitrogen flux (the amount of nitrogen leaving the edge of a field) if not managed properly. The 2007 EISA mandates the production of 136 billion liters of biofuels by 2022, of which 57 billion liters is corn ethanol and 80 billion liters is from sources other than corn, such as cellulosic sources and biodiesel. To meet the mandates, the agricultural sector is expected to increase corn production by increased chemical and fertilizer applications on the fields and less crop rotation with soybeans [7]. Shifting the crop rotation from the conventional corn-soybean to continuous corn would significantly increase nitrate-N runoff to surface water, because continuous corn systems require a large fertilizer input [8]. The use of biofuels in the transportation sector is a very small, but growing fraction of the total fuel consumption, approximately 4.5% in 2011 [9]. This small percentage consisted of 49 billion liters of corn ethanol that were produced using 41% of the corn production [10]. Increased biofuel usage will increase nonpoint source pollution (e.g., fertilizer and pesticides) runoff to waterbodies, as well as other less direct environmental impact [11,12,13]. A study by [14] to assess the impact of biofuel crop production on the formation of hypoxia region in the Gulf of Mexico found that meeting the 2022 biofuel mandate will increase the average nitrate-N output by 300,000 to 750,000 metric tons depending on biomass sources used for fuel. This represents approximately 23.8% to 60% of the average annual total nitrogen flux to the northern Gulf of Mexico from 2005 to 2009. Even though scenarios where ethanol is produced using only cellulosic crops had lower average NO_3_ fluxes than those of corn for the production of ethanol, the range of nitrate fluxes for all scenarios highly overlap, which indicates the uncertainty in achieving any specific scenario [14]. Cellulosic ethanol scenario had lower NO_3_ fluxes than those of corn ethanol, because the nitrogen application for cellulosic crops is roughly half of the nitrogen needed for corn [15], and grasslands are not tilled, which reduces runoff [16]. Farmers can increase corn production by not only increasing fertilizer application, but also increasing corn acreage. The work in [17] showed that a 14.4% increase in corn acreage would result in a 5.4% increase in total nitrogen loads and in a 4.1% increase in total phosphorus. 

In order to capture the benefits of using biocrops to produce biofuels, their impact on the environment must be addressed and minimized. As mentioned above, agricultural practices in the upper Mississippi region have contributed to nonpoint source pollution, mainly nitrogen, causing a decline in water quality downstream. Excess nutrient runoff can lead to eutrophication of water bodies and can cause hypoxic conditions (dissolved oxygen <2 mg/L). The hypoxia zone in the northern Gulf of Mexico occurs annually due to the overgrowth and decomposition of organic matter, affecting the natural functions of the ecosystem and threatening commercial and recreational gulf fisheries valued at $2.8 billion annually [4]. The size of the hypoxia zone varies annually (the long-term average is 13,825 km^2^); thus, the Mississippi River/Gulf of Mexico Watershed Nutrient Task Force established a goal to reduce the size of the hypoxia zone to 5000 km^2^ by 2015. However, there is little evidence of progress in reducing the size of the hypoxic zone, as the current five-year average (2006 to 2010) is 17,300 km^2^ [4]. The increased demand for biofuels will results in more demand for corn (more fertilizer being applied), which would further impact the water quality. 

Establishing riparian buffers between crop fields and streams has proven to be an effective management practice for reducing nitrogen flux from anthropogenic inputs. Nitrogen species are transformed by fixation, immobilization, soil storage and groundwater mixing [18] and removed from the system by denitrification [19]. The effectiveness of riparian buffers depends on their ability to reduce the speed of runoff and to decrease the nitrogen flux in surface and subsurface runoff. Factors, such as buffer width, landscape and hydrogeomorphic characteristics, play a major role in the nitrogen removal processes [20]. Studies have shown that riparian buffers can remove nutrients and effectively limit sediment runoff from fields [21,22,23,24,25,26]. The work in [23] measured the effectiveness of using switchgrass strips as buffer zones and found that total nitrogen was reduced by 80%. The work in [27] estimated the nitrogen immobilization rates in switchgrass sites to be an average of 16 kg·N per ha. Nitrogen management efforts could benefit from targeting watersheds with the highest nitrogen fluxes; however, using models to predict nitrogen fluxes yields uncertainties in the model predictions, which must be addressed. A meta-data study by [24] summarized the results from 88 peer-reviewed studies on the effects of riparian buffer width on nitrogen removal. The influent concentrations ranged from 0.02 mg/L to 375 mg/L. The study found that riparian buffers have a mean nitrogen removal effectiveness of 67.5% and that a buffer width larger than 50 m was more consistent in removing significant mass of nitrogen. The study also found that the removal effectiveness varies nonlinearly with width, but no significant removal differences were found among vegetation type.

The objectives of this study are related to estimating nitrogen load resulting from the increased energy crop production used to meet the EISA mandates in order to address the following three important questions: (1) What is the contribution of biofuels to nitrogen loading in the Mississippi River watershed?; (2) Does switching to cellulosic ethanol drastically change the total nitrogen loading when compared to corn ethanol?; (3) Which catchments have high nitrogen flux resulting from meeting the mandates and warrant future investigation into nitrogen mitigation or management strategies? We achieved these objectives by calibrating a geospatial nitrogen model and then applying model results to future scenarios of biofuel production. Section 2 details the model calibration and the datasets used in our analysis. Section 3 discusses the results, and Section 4 presents a summary and concluding remarks.

## 2. Materials and Methods

### 2.1. Estimating Total Nitrogen Runoff

The SPAtially-Referenced Regressions On Watershed attributes (SPARROW) by [28] is a watershed modeling technique that uses a hybrid statistical/mechanistic approach to estimate pollution sources and the transport of pollutants to surface waters. SPARROW utilizes statistical methods to explain the water quality measurements (constituent mass or load) taken at a given stream in relation to upstream sources and watershed properties, such as precipitation and land use, soil properties that are thought to influence the transport of pollutants to streams [29]. The water quality data are used to estimate the mass annual load of pollutants that enter a stream at each monitoring station site. Geospatial data are then used to relate watershed attributes with load estimates. On a national scale, SPARROW was applied to estimate national nitrogen and phosphorus models to predict the flux of nutrients to the Gulf of Mexico [30]. On a regional scale, SPARROW was used to estimate nutrients in streams located in New England [31]. The system boundary is the whole Mississippi River watershed, which makes SPARROW advantageous for the study for several reasons. First, SPARROW is a hybrid model that combines statistical approaches to estimate parameters specific to a given region with a mechanistic functional form of the model (mass balance). Second, it utilizes Geographic Information System (GIS) data allocated to individual stream catchment areas; thus, SPARROW can be applied to a large region without assuming uniform properties for the whole region Third, SPARROW relies on a detailed stream reach network to which data on stream characteristics are spatially referenced. The model uses a mass balance approach to quantify the long-term supply, transport and fate of nutrients in streams and watersheds. In general, the load leaving the reach is the sum of (1) load generated within the upstream reaches and transported to the given reach and (2) the loads generated within the reach’s own watershed [29]. The loads (dependent variables) are evaluated as a nonlinear function of the independent variables, such as nitrogen sources (agriculture, point sources, atmospheric deposition), land to water delivery (precipitation, soil permeability, land cover) and decay processes (hydraulic retention time and settling) (refer to [29] for further details regarding SPARROW). SPARROW is applied in this study to estimate the spatial distributions of total nitrogen, sources of the nitrogen and the delivery of nitrogen to Gulf of Mexico. The results of the model can be used to identify the variables that are significant predictors of nitrogen levels in streams and to evaluate the impacts of various nitrogen inputs on water quality. The major difference between this study and other SPARROW studies is that it estimates the contribution of individual crops (rather than aggregate) and various land uses to nitrogen loading. Furthermore, the nitrogen model is estimated for the Mississippi River basin to include the major crop regions and to assess the nitrogen flux to the northern Gulf of Mexico. The estimated nitrogen model is then used to predict total nitrogen loadings from energy crop production. 

Crops considered in this study are corn/soybeans, hay (excluding alfalfa), switchgrass and an aggregate of all other crops, including alfalfa. The original approach was to model estimated runoff from corn and soybeans separately. However, the majority of corn and soybeans is grown on rotation fields, and there are no data to describe which crop was grown on such fields at a specific point in time (year); also, more than 80% of corn and soybeans are produced on rotation fields [32]. The corn and soybean variable explains the average runoff leaving corn/soybean rotation fields. Hay and other herbaceous plants are used here as a surrogate for cellulosic plants, such as switchgrass, to produce cellulosic ethanol. Hay is used as a surrogate cellulosic plant for the following reasons: (1) there is not enough switchgrass production in the U.S. to explain a correlation between its nitrogen loading and production; and (2) there is no clear primary source of cellulosic ethanol. At any given fertilization rate, the yields of hay and switchgrass are comparable to those of switchgrass; hay is used here to provide an initial approximation of the nitrogen flux as a result of producing cellulosic ethanol. To estimate the nitrogen flux from switchgrass, an *ad hoc* approach was used in the model’s predictions by replacing the hay fertilization rate and yield with that of the switchgrass fertilization rate and yield reported in the literature (see Section 3.2).

**Figure 1:**
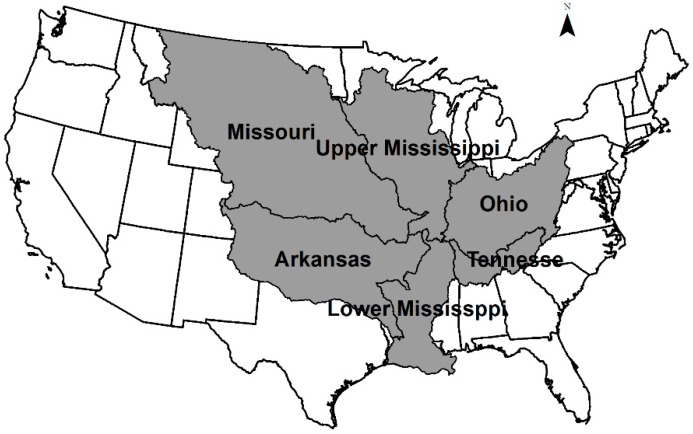
River basins used in SPARROW.

The area for the SPARROW model includes the Upper and Lower Mississippi River, Ohio River, Missouri River and Arkansas River basins (Figure 1). For this study, a regional total nitrogen model was calibrated for year 2002, the selected period coinciding with the latest data compiled by the United States Geological Survey (USGS) for SPARROW purposes (USGS, 2013). In order to assess the nitrogen flux from the EISA mandates, the following scenarios are considered; EISA 2015 and 2022 mandates and hypothetical scenarios, such as 2022 all corn ethanol, 2022 all cellulosic ethanol and 2022 all soybeans biodiesel. The scenarios will be compared based on two criteria, total nitrogen flux and land requirements. These projections are based on 2002 land use; while it is impossible to determine where corn and soybeans will be grown in the future, it is assumed that the future demands will be met through increased production in the Mississippi River basin. 

### 2.2. Load Estimation 

The nitrogen load estimates (dependent variables) were obtained from a study by [33] that estimated the long-term mean annual nitrogen loads (de-trended to 2002) for 2739 monitoring stations throughout various Major River Basins (MRBs). The authors compiled water quality data from federal, state and local agencies and from selected universities. Most water-quality data were obtained from the National Water Information System (NWIS) and the U.S. Environmental Protection Agency’s STOrage and RETrieval (STORET) database. The authors estimated the detrended load estimates based on two models described by [34] (refer to [32] for further details regarding the load estimated). 

### 2.3. Hydrologic Network 

This study utilized the digital hydrologic streams network that was developed by [35] to support SPARROW models within selected regions of the United States. This network is used by SPARROW to define surface-water flow paths that connect nitrogen sources and stream catchments with observations of water quality at downstream monitoring stations [29]. It is a 1:500,000-scale Major River Basins Enhanced River Reach File 2.0 (MRB_E2RF1) reach network for the conterminous U.S. The reach network includes characteristics that describe the morphology and hydraulic conditions of stream reaches, such as mean discharge, mean velocity, reach length and travel time. The network also includes reservoir and lake properties, such as surface area and discharge. For this study, only the reaches located in Missouri, Mississippi, Ohio and Arkansas River basins were used. Incremental catchments for the 27,982 stream reach basins mentioned above were delineated from 100-m digital elevation models [35].

### 2.4. Input Sources, Transport and Decay Variables. 

Nutrient sources considered in this study included point sources (sewerage, commercial and industrial dischargers), land cover classes (such as developed, forested and shrub lands), fertilizer applied to agricultural land and atmospheric deposition of nitrogen (natural and anthropogenic). Some variables, such as developed lands, may serve as a measure of various nonpoint urban sources that enter streams through runoff from surfaces and inflows from groundwater in urbanized catchments, for example lawn fertilizers and septic tanks. Data regarding point sources were obtained from [36]; the results of their study were used as inputs for the SPARROW model in this study. The authors estimated the nutrient loads from point sources, such as sewage treatment, commercial and industrial plants, using data from the USEPA Permit Compliance System (PCS) national data warehouse [37].

The atmospheric nitrogen deposition data used as input to SPARROW are based on the use of wet deposition measurements at the National Atmospheric Deposition Program (NADP). Nitrogen deposition data were obtained from [38]. The authors estimated the long-term annual nitrogen deposition (detrended to base year 2002) and allocated it to MRB_E2RF1 catchments. These estimates were calculated from annual measurements (1990 to 2005) at 186 stations throughout the United States. The atmospheric nitrogen estimated by SPARROW would be expected to reflect regional nitrogen sources, such as vehicle emissions and urban lands [39].

Data on land cover areas throughout the conterminous United States used in the model were obtained from [40]. The authors allocated the National Land Cover Database (NLCD) 2001 [41] land use categories, which include water, developed, barred, forest, scrubland, herbaceous, planted/cultivated and wetland, to MRB_E2RF1 catchment areas. The land use/land cover input areas used for this study are urban, barren, scrubland and herbaceous and forested areas. The nutrient sources from urban land area serve as a measure of different urban sources, such as inputs from septic systems and surface runoff from fertilized land, such as lawns and parks and other urban sources.

Data on annual nitrogen inputs to various crops used in this study were obtained from [42]. The authors estimated the fertilizer and manure inputs from 2002 sales and expenditures data from the Association of American Plant Food Control Officials and the U.S. Census of Agriculture [43] and allocated to MRB_E2RF1 catchments by the fraction of the catchment’s agricultural land [42]. Farm fertilizer sales serve as a direct measure of commercial fertilizer use and intensity of farming practices. Manure data included inputs from confined (animal feeding operations for cattle, poultry and dairy operations) and unconfined (farm, pasture and range livestock operations) sources.

Air temperature data for year 2002 averaged from minimum and maximum daily temperature values were obtained from the Parameter-elevation Regressions on Independent Slopes Model (PRISM) digital data network (PRISM, 2012) and allocated to MRB_E2RF1 catchments by [44,45]. The 2002 annual precipitation data were obtained from the Parameter-elevation Regressions on Independent Slopes Model (PRISM) digital data network [46] and allocated to MRB_E2RF1 catchments by [47]. Mean soil permeability was obtained from the State Soil Geographic (STATSGO) Data Base digital data [48] and allocated to MRB_E2RF1 catchments by [49]. 

### 2.5. Scenarios

In order to assess the nitrogen flux from the EISA mandates, the following scenarios are considered: EISA 2015 (78 billion liters) and 2022 (136 billion liters) mandates, where the biofuels consist of corn ethanol, cellulosic ethanol and biodiesel. A set of hypothetical scenarios is also considered: corn ethanol 2022, where the mandate is met with ethanol made from corn, soybean biodiesel 2022, where the mandate is met by biodiesel made from soybeans, and cellulosic ethanol 2022. As mentioned earlier, hay and switchgrass are used in this study as crops to produce cellulosic ethanol; thus, there are two “sub” scenarios for cellulosic ethanol based on the crop selected. The scenarios will be compared based on two criteria, total nitrogen flux and land requirements. These projections are based on 2002 land use; while it is impossible to determine where corn and soybeans will be grown in the future, it is assumed that the future demands will be met through increased production in the Mississippi River basin.

### 2.6. Nitrogen Management Strategy

A common strategy for nitrogen mitigation is to establish a buffer zone along stream and river corridors. The buffer zone is typically comprised of vegetated areas and serves the purpose of intercepting nutrient laden runoff before it reaches the stream. Important considerations in buffer design are where to construct the buffer area and the amount of nitrogen to be managed. Delineating the exact location of riparian buffer areas for the upper Mississippi River is beyond the scope of this study. Instead, an attempt was made to identify the catchments with high nitrogen effluent concentrations and flux (high impact catchment areas) resulting from meeting the mandates and the associated uncertainties with the fluxes. The high impact catchment areas are the catchments that produce 25% of cumulative nitrogen flux generated from EISA biofuels mandates. For simplicity, it is assumed that the riparian buffers intercept the nitrogen flux generated in the catchment before it is discharged in the stream. This is an arbitrary management scenario; however, it will provide a preliminary assessment on reducing nitrogen flux from the biofuels. It is important to note that the flux and concentrations are based solely on biofuels production and do not take into account the other sources of nitrogen. The high impact catchments were identified by using SPARROW’s nitrogen flux prediction in each stream. Since it was difficult to predict the future locations of crops fields, 2002 land use data are used to approximate nitrogen flux for the mandates for each catchment area. The total nitrogen flux estimated for 2015 and 2022 mandates was allocated to stream catchments based on the fraction of crops (corn, soybean, hay) relative to the total in the study region. The concentration of nitrogen effluent was calculated by dividing the total nitrogen flux by the stream flow in the catchment. The predicted fluxes from SPARROW are dependent on the multiplicative error term that represent other variables that are not included in the model, making the nitrogen predictions subject to uncertainties. These uncertainties were assessed using the 90% confidence intervals estimated by SPARROW using bootstrapping procedures based on resampling the data to generate 200 sets of models; for more details, refer to [29].

## 3. Results

### 3.1. Calibration Results

Model coefficients (Nonlinear Weighted Least Squares NWLS estimates) and the nonparametric bootstrap mean estimate of the coefficients results for the SPARROW model developed for this study, including parameter estimates, standard errors and *p*-values, are summarized in Table 2. The model was calibrated using 1003 observations from monitoring stations throughout the region. Since the input data are highly uncertain and the model describes a large watershed, a *p*-value of <0.1 was used to make certain that the probability of obtaining an estimate was not too constricting. Furthermore, many studies used this *p*-value [30,33]. The total nitrogen model is as a function of seven nitrogen sources (nitrogen applied to corn/soybeans, hay, other crops, atmospheric deposition, point sources, urban land and forests); two land-to-water delivery factors (precipitation and air temperature); and nitrogen decay (due to denitrification, biological uptake and sedimentation) factors, which are a function of retention time in streams and reservoirs. 

The model results in Table 2 only include significant variables; other variables were removed from the model because they were highly insignificant, and removing them improved the model’s fit. Most coefficients (except other crops) were significant (*p* < 0.1), indicating that each of the nitrogen inputs, land-to-water and decay variables are important in explaining the variation and the distribution in the measured loads at monitoring stations. Even though the variable “other crops” was insignificant, it was left in the model, because it is believed that other crops could be a significant source of nitrogen in subregions not dominated by corn and soy. Most of the standard errors are small compared to the magnitude of the coefficient, which indicates their resemblance to the overall population (except those for other crops and forests). Table 2 also shows that NWLS and bootstrap estimates are close in value, which indicates the stability of the model coefficients and confidence in the NWLS results, which rely on the parametric estimation of the coefficients. Only the NWLS coefficients are used for further applications and analysis in this study.

The total nitrogen model explained approximately 93% (adjusted *R*^2^) of the spatial variability in the log-transformed annual nitrogen load (Table 2). The *R*^2^ value for SPARROW models is generally high because of the strong relation between drainage area and annual discharge [29]. After normalizing the annual nitrogen load for drainage area (referred to as yield hereafter), the model explained ~87% of the variability (Table 2). The root mean square error (RMSE), which describes the accuracy of the model predictions (nitrogen loads), was 0.57. The model was evaluated for evidence of regional prediction biases by visually inspecting each of the calibration sites’ studentized residual map to identify the large over and under predictions. Studentized residual >3.6 or <−3.6 are considered outliers and require more investigation [29]. The majority of residuals (95%) fall between −2.26 and 2.03; there are eight sites where the residuals values are considered outliers. The Shapiro–Wilk test (Table 2) indicates that the normalized residuals are weakly normal (*p* < 0.1), and according to [29], the model is robust enough to utilize weakly normal residuals. 

The coefficients for the nutrient sources provide an estimate of the fraction or quantity of each input delivered to streams [29]. The point sources’ coefficient should be near one, since they discharge directly to streams and are unaffected by the land-to-water variables. However, the SPARROW estimate for point sources in this study was approximately 0.72, meaning that the point sources were underestimated, a result similar to [50]. The work in [29] points out that estimates should approximate one if all sources are accounted and losses are accurately described by the land-to-water variables. Thus, the point source estimate was less than one in this study and could be a result of using land to water variables that are not a true representation of the actual processes. As expected, nonpoint-source estimates are substantially smaller than one (Table 2) because they have been subjected to natural processes (denitrification plant uptake and/or remain in the soil). The source coefficients indicate that 19% of the nitrogen applied to corn/soy fields and 8.5% of the applied nitrogen to hay fields run off to nearby streams. The differences in runoff rates is due to agricultural practices and fertilization rates, as nitrogen application for hay/switchgrass is roughly half of the nitrogen needed for corn [15], and grasslands are not tilled, which reduces runoff [16]. The results in Table 2 were compared to the results from [14,51], and the results are comparable to the results presented in this study. The authors have estimated that 24% of nitrogen applied to corn and soybean fields leaves as runoff, while this is 13% for switchgrass. The negative sign of the temperature coefficient indicates that there is less nitrogen reaching the streams in regions with high air temperature, which could be attributed to increased biological activities (denitrification and plant uptake) [29,30]. The wetlands coefficient is also negative, which indicates nitrogen decay in wetlands. Precipitation, on the other hand, increases the nitrogen delivered to streams due to the increase in surface runoff [29,30]. The aquatic loss coefficients have a positive sign, which indicates that nitrogen is decayed as it travels downstream and into reservoirs [29,30].

### 3.2. Model Predictions

The second objective of this study was to estimate the amount of nitrogen flux resulting from EISA mandates and to compare them to other hypothetical scenarios. By year 2022, approximately, biofuels will be produced from corn (42%) and cellulosic crops (44%), respectively, and the rest is biodiesel from various sources. While it is still unclear what type of cellulosic crop(s) will be used in the future, switchgrass seems to be the main focus of many studies [12,14,52,53,54]. To estimate the nitrogen flux resulting from meeting the renewable fuel mandates, the biofuel volumes for the scenarios mentioned above were converted to nitrogen inputs using crop fertilization rates, crop yields and crop-to-fuel conversion factors (Table 3). The nitrogen inputs were then used in SPARROW to predict the nitrogen runoff using the calibrated model. All cellulosic ethanol is assumed to be produced from hay, and since the mandates did not specify which crops are to be used for biodiesel production, for this study, it is assumed that biodiesel is produced from soybeans, an assumption [14] used in their study. While this is a hypothetical scenario, the rationale for it is to assess the total nitrogen flux from using soybeans as a source of biodiesel. 

**Figure 2:**
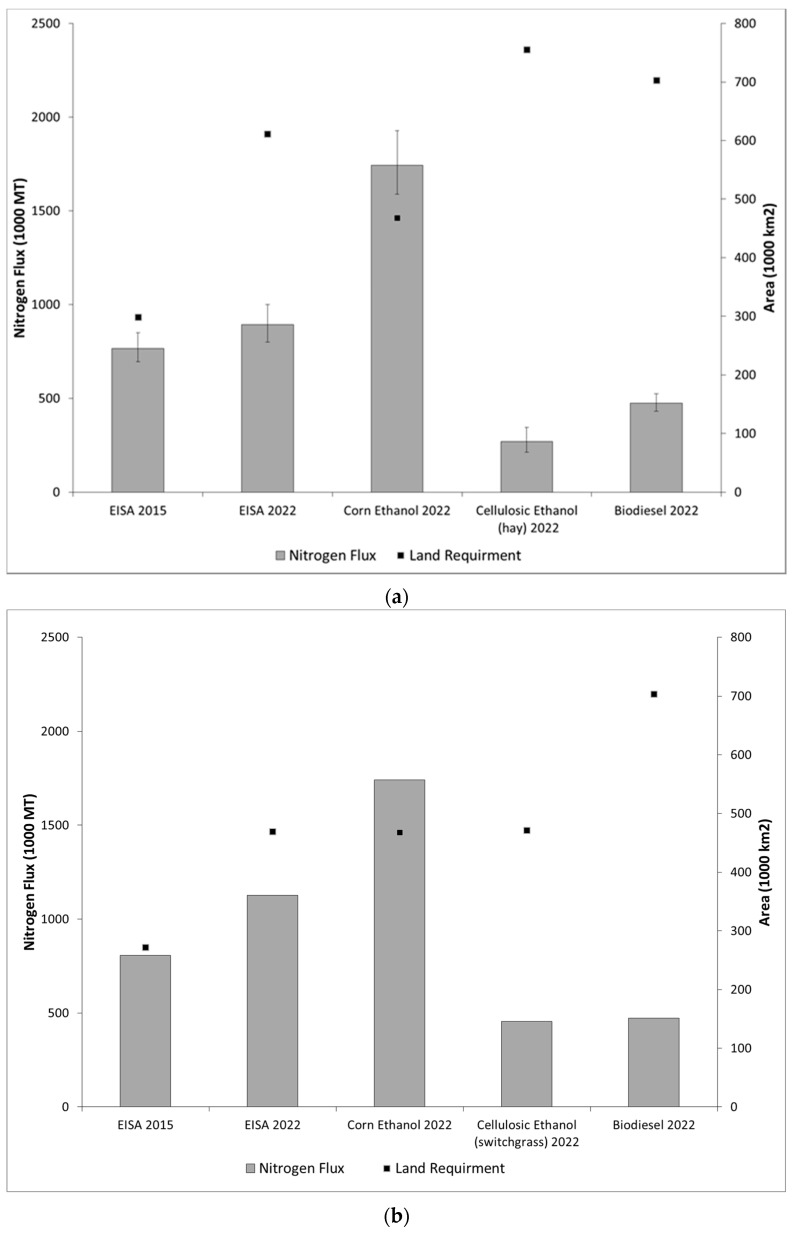
Total nitrogen flux and land requirements for different biofuels scenarios. Nitrogen fluxes using hay (**a**); nitrogen fluxes using switchgrass (**b**).

**Figure 3:**
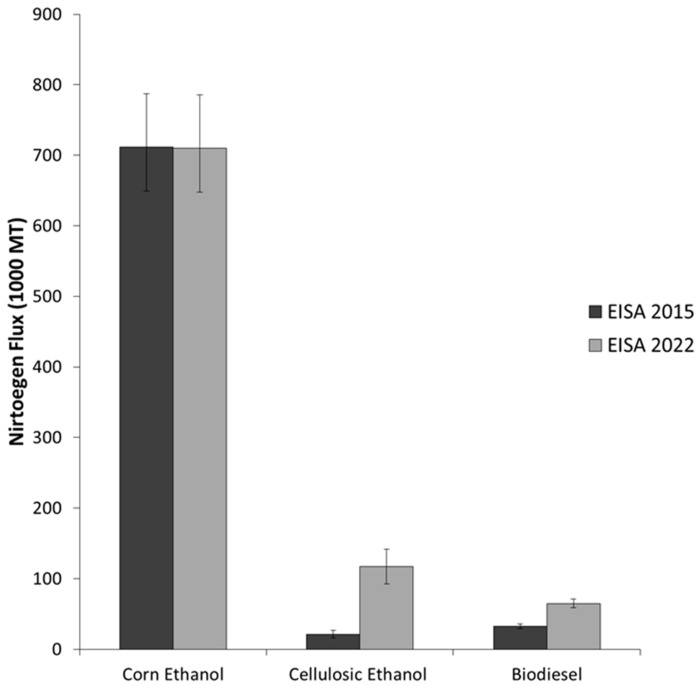
Nitrogen flux by fuel type for EISA mandates 2015 and 2022.

Figure 2 shows the estimated annual total nitrogen flux resulting from meeting the different biofuel scenarios and the different areas required by each scenario. The area for each scenario was calculated by multiplying the mandate volume times the inverse of the conversion factors and yields. As expected, meeting the 2022 ethanol mandate using only corn produced the largest nitrogen flux (1.7 million metric tons (MT)), whereas the all cellulosic ethanol scenario produced 270 thousand MT of nitrogen. The total nitrogen flux from meeting the 2015 and 2022 EISA mandates is 765.3 and 892.3 thousand MT, respectively. This result compares reasonably well to [14], who estimated nitrate-nitrogen for the same years (2015 and 2022) to be 580 and 600 thousand MT. Part of the difference is likely to the fact that [14] did not include biodiesel in their estimates. Meeting the mandates using only cellulosic ethanol or biodiesel requires large areas of land due to low hay and soybeans yields (mass/area) compared to that of corn. The 90% confidence intervals (CI) (shown as error bars in Figure 2a) indicate the reliability of the predictions and reflect their spatial variability. After investigating the 90% CI, it was concluded that the 2022 nitrogen loads from biofuels is not different from those of year 2015, and that is mainly due to the large variability of corn ethanol estimates. Figure 3 shows that loads from corn ethanol do not change due to capping corn ethanol at 57 billion liters per year (712 thousand metric tons or approximately 80% of the total nitrogen load in 2022); the rest of the nitrogen flux results from the cellulosic ethanol production in both scenarios. The annual total nitrogen load from cellulosic ethanol in 2022 is significantly less than that of corn ethanol (117.5 and 712 thousand metric tons, respectively), even though the volume of fuel mandated is roughly the same. This is due to the low fertilization rate compared to corn (Table 3). Nitrogen load from soybeans is very small, because the biodiesel mandate is small relative to ethanol fuels. It is important to note that hay fertilization rate (0.0028 kg·N/m^2^) and yield (0.5 kg/m^2^) were used to estimate the nitrogen flux and land requirements for cellulosic ethanol; however, switchgrass is produced using higher fertilization and yield (Table 3). 

**Figure 4:**
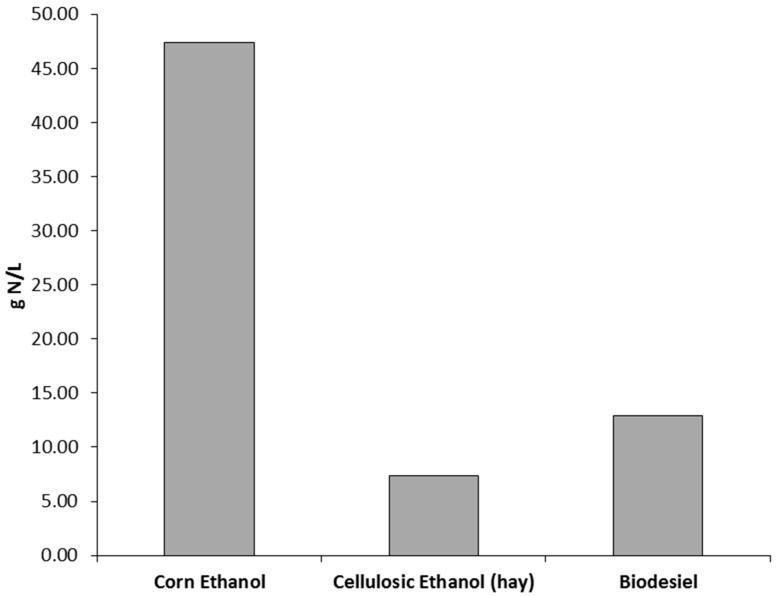
Total nitrogen flux and land requirements per volume of fuel.

The nitrogen flux from switchgrass ethanol mandate (Figure 2b) was estimated as follows: (1) the mass of switchgrass for cellulosic ethanol was calculated using data in Table 3; (2) the area required for switchgrass was calculated by multiplying the mass by the yield (0.9 kg/m^2^) described by [58]; (3) the area was multiplied by the fertilization rate (0.0112 kg·N/m^2^) to calculate the total nitrogen required for switchgrass, then multiplying it by hay’s nitrogen runoff coefficient estimated by SPARROW. While using a higher fertilization rate results in more nitrogen flux than hay, the increased yield results in a smaller area requirement for switchgrass. Normalizing the total nitrogen estimates for fuel volume, the results indicate that the nitrogen fluxes are 47.3 g/L, 7.3 g/L and 12.9 g/L for corn ethanol, hay ethanol and soybeans biodiesel, respectively (Figure 4). The work in [59] approximated the nitrogen fluxes per volumes of biofuels where the nitrogen flux was 45 g/L for corn ethanol and 35 g/L for soybean biodiesel, but no estimate for cellulosic (hay) ethanol. The discrepancies between the results of the two studies are mainly due to the inputs used in the estimation, such as yield, fertilization rates and conversion factors. 

**Figure 5:**
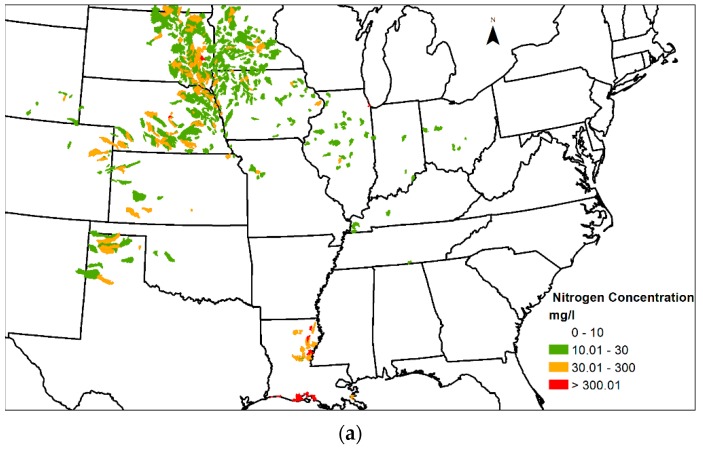

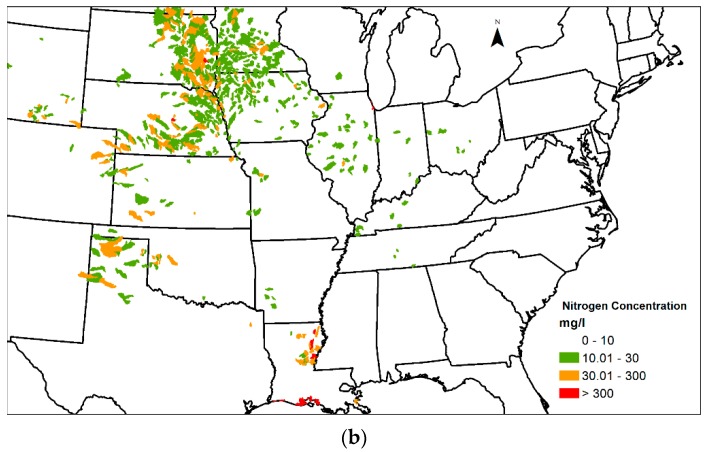
(**a**). Distribution of nitrogen concentration per catchment area for the 2015 mandate. (**b**) Distribution of nitrogen concentration per catchment area for the 2022 mandate.

**Figure 6:**
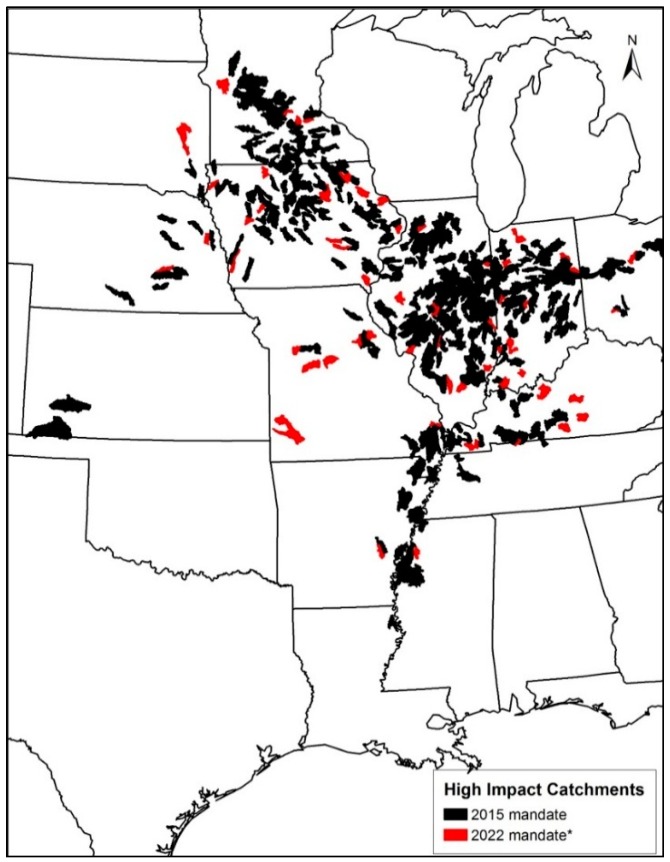
High impact catchment areas. * Additional areas required by the 2022 mandate.

Figure 5 shows the spatial distribution of nitrogen-producing catchments and their associated concentrations. Both Figure 6a,b show a similar catchment distribution, where the majority of catchments have a concentration of 10 mg/L and below. The figures also show that catchments with concentrations of 30 mg/L and above are clustered in the region between the Missouri and the Upper Mississippi River basins. As mentioned above, these concentrations only reflect the biofuel production in the catchments; thus, the purpose of Figure 5 is to show where biofuel production will likely impact the water quality at the catchment level. The mean removal effectiveness of riparian buffers is 67.5% [24], with an effective concentration up to 300 mg/L (based on the highest concentration reported in the study). That means that the riparian buffers are capable of reducing the concentrations in most catchments to below 100 mg/L. 

**Figure 7:**
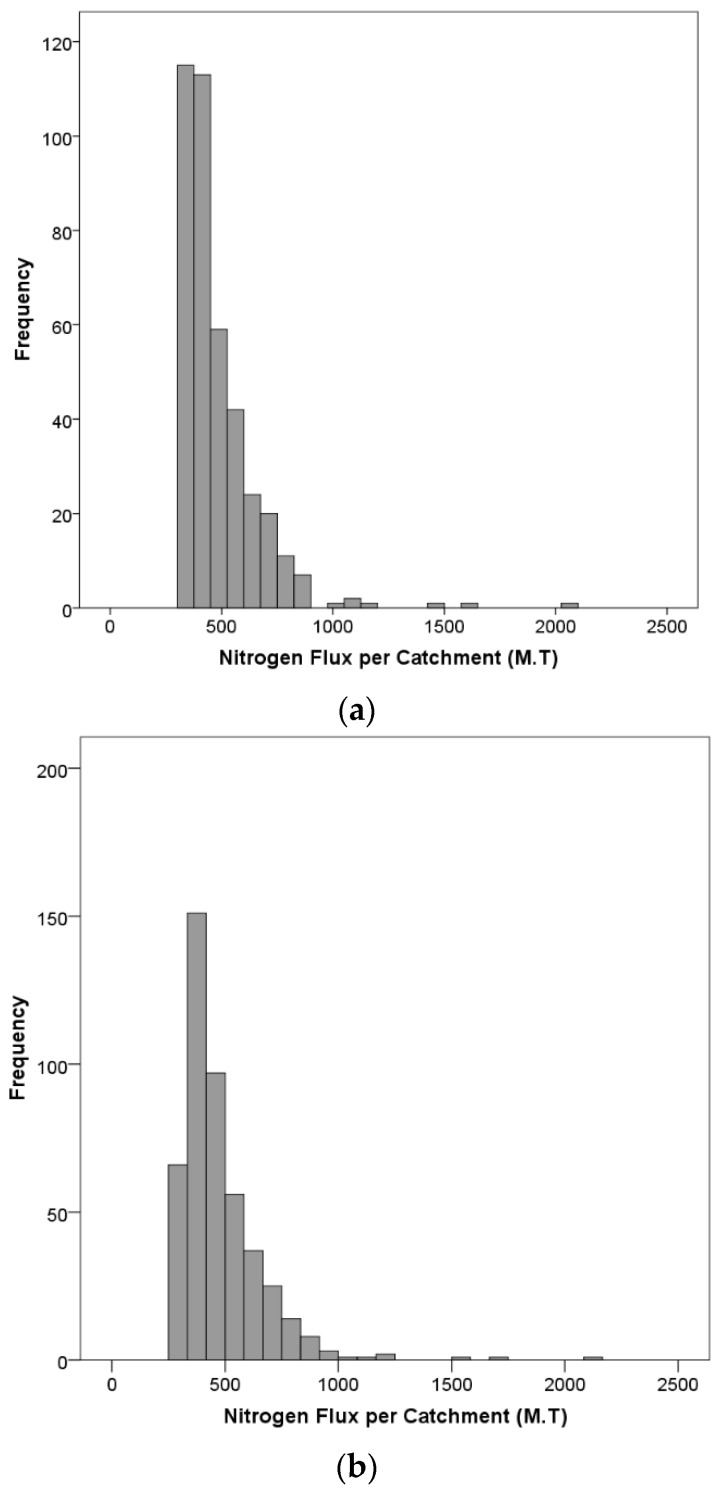
Distribution of nitrogen flux per catchment area for the 2015 mandate (**a**) and for the 2022 mandate (**b**).

From a nitrogen reduction objective, it is important to locate the catchments that produce large fluxes of nitrogen (high impact catchments). These catchments that may require nitrogen mitigation strategies (other than riparian buffers) associated with the 2015 and 2022 mandates are shown in Figure 6. Catchments were ranked based on nitrogen fluxes from highest to lowest, and then, the catchments that contributed 25% of the cumulative nitrogen flux for the 2015 mandate are shown in black. Red indicates the additional catchments for the 2022 mandate. The highest nitrogen fluxes are generated in catchments located in the Upper Mississippi, Lower Mississippi and the Missouri River basins, which closely coincide with intense corn and soybean farming. On average, the total nitrogen fluxes from these catchments are approximately 191.3 and 222.8 thousand MT for the 2015 and 2022 mandates, respectively (Table 4). The average flux remained almost the same for both years, because the majority of nitrogen fluxes in both years is a result of corn ethanol demand, which remained fixed at 57 billion liters per year. The distribution of the nitrogen fluxes for years 2015 and 2022 is positively skewed with the majority of the fluxes range from 250 to 1000 MT (Figure 7). This information can assess a management agency in the overall buffer design, especially in determining the width of the buffer zones. 

The high impact catchments for year 2015 covered an area of 179.1 km^2^, while the area for year 2022 was 202.4 km^2^. The increase in area can be explained by the increased demand for hay to meet the cellulosic ethanol demand by the 2022 mandate. Given the estimated nitrogen flux, the theoretical potential for switchgrass production in riparian buffers was calculated assuming 100% capture of nitrogen, which was translated into ethanol production (Table 4). These calculations could be used as a reference for the maximum ethanol production potential from riparian buffers. The annual volume of ethanol (theoretical) that could be produced from switchgrass riparian buffers is approximately five and 5.9 billion liters for mandates 2015 and 2022, respectively, which could present an economic opportunity for farmers to manage nitrogen using riparian buffers.

**Figure 8:**
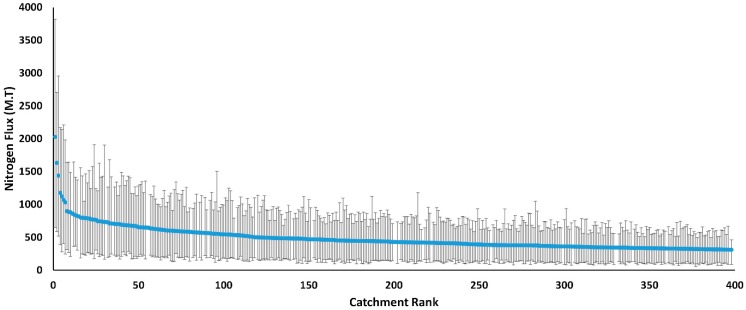
Nitrogen flux as a function of catchment rank.

One important aspect in management decisions is the uncertainty in the model’s predictions. To assess these uncertainties in nitrogen predictions, the 90% confidence intervals of the prediction were plotted against the rank of the catchment based on its nitrogen flux (one being the highest flux) (Figure 8). Furthermore, the differences in the nitrogen flux from the majority of low ranking catchments is small, especially given the overlap in their confidence intervals, which makes it difficult to reliably rank them based on their nitrogen flux. The wide range of confidence intervals could present a challenge from a nitrogen management point of view when it comes to designing buffer zones and also the potential switchgrass that could be grown. 

In summary, the results from modeling the different biofuels scenarios indicate that meeting the biofuel goals mandated in the 2007 EISA will increase the nitrogen flux in the Mississippi River watershed mainly due to increased feedstock production to meet the growing demand. Cellulosic (hay/switchgrass) and soybean biomasses show a promising potential in reducing the nitrogen flux by a significant amount when compared to corn scenarios. However, the land required to grow cellulosic biomass and soybeans to meet the 2022 mandate is approximately two-fold the land used to grow corn and soybean (total production) in 2015 [60]. While the feasibility of such land use is beyond the scope of this study, it is apparent from the results that there is a tradeoff between nitrogen runoff and land use. Thus, in order to reduce and nitrogen flux resulting from the EISA mandates (Table 1), nitrogen mitigation options, such as using buffer zones, are potentially effective [18,22,23,24,27,61]. The results show that the nitrogen flux from the catchment that contributes 25% of the cumulative nitrogen flux could be 191 metric tons and 223 metric tons a year for the EISA 2015 and EISA 2022 scenarios, respectively. However, reducing the nitrogen flux using the buffer zone size of the area of interception and the nitrogen removal effectiveness has an average of 67.5% [48]. The results from this study (Table 4) show that the area to be intercepted is approximately 179 thousand km^2^ and 232 thousand km^2^ for EISA 2015 and 2022, respectively. These estimates, however, could be improved by using high resolution geospatial catchment data (1:24,000) [62], rather than the current catchment resolution of 1:500,000.

## 4. Discussions 

One of the main objectives of the Energy Independence and Security Act (EISA) is to promote energy independence through mandating that U.S. transportation fuel contains domestically-produced biofuels, mainly ethanol. The majority of the energy consumed in the U.S. comes from fossil sources; only 11% of the total energy consumption in 2011 is classified as renewable energy (biomass, geothermal, wind, solar and conventional hydropower). Biofuels account for a small portion of the total U.S. energy sources, but have significant environmental impact. Increased biofuel usage will increase nonpoint source pollution (e.g., fertilizer and pesticides) runoff to waterbodies, as well as other less direct environmental impacts [11,12,13,14]. The hypoxia zone in the northern Gulf of Mexico occurs annually due to the overgrowth and decomposition of organic matter, affecting the natural functions of the ecosystem and threatening commercial and recreational gulf fisheries valued at $2.8 billion annually [4]. The size of the hypoxia zone varies annually (the long-term average is 13,825 km^2^); thus, the Mississippi River/Gulf of Mexico Watershed Nutrient Task Force established a goal to reduce the size of the hypoxia zone to 5000 km^2^ by 2015. However, there is little evidence of progress in reducing the size of the hypoxic zone, as the current five-year average (2006 to 2010) is 17,300 km^2^ [4].

The objective of this study was to use the SPAtially Referenced Regressions On Watershed attributes (SPARROW) modeling method to estimate nitrogen fluxes in the Mississippi River basins as a result of EISA biofuel mandates. The model was estimated to reflect 2002 land use, climatic conditions and basin characteristics. This study was limited to assessing the nitrogen flux associated with biofuel production. There is an array of environmental impacts that must be taken into consideration, such as water demand, pesticide use and other nutrients, such as phosphorus. It must be noted that the model assumptions and input present (crop yields, fertilization rates, conversion factors and agricultural management practices) current values and were not adjusted to reflect future (year 2022) improvements in the model inputs. These assumptions were made due to limited information on the future trends in the values used for model prediction, as most projections are merely time extrapolations, which are highly uncertain. In addition, the SPARROW model used in this study is a steady state model, which does not address seasonal variations and changing weather patterns from year to year. 

The scenario results show that biofuel production may result in an increase of nitrogen flux to the northern Gulf of Mexico from 270 to 1742 thousand MT. That is an increase of 21% to more than 100% over the total nitrogen flux estimated by the [4]. Using all cellulosic (hay) ethanol or biodiesel to meet the 2022 mandate is expected to significantly reduce nitrogen flux; however, it requires approximately 25% more land than the land needed in EISA-specified 2022 scenario. As mentioned above, hay is used as a surrogate cellulosic plant because there is not enough switchgrass production in the U.S. to explain a correlation between its nitrogen loading and production, and there is no clear primary source of cellulosic ethanol. However, it is important to note that hay underestimates the nitrogen flux prediction because the fertilization rate is lower than that of switchgrass. Producing ethanol from switchgrass rather than hay results in three-times more nitrogen flux, but requires 43% less land. The all-corn ethanol for the 2022 scenario mandate is expected to double the nitrogen flux when compared to the EISA-specified 2022 scenario, however, it will require less land area. 

## 5. Conclusions

This study does not recommend one scenario over the others, because evaluating the cost and benefits of each scenario is beyond the scope of this study. The results can be used by the policy makers to access the different alternatives in meeting the EISA mandates, and choosing the best scenario will depend on the value of the resources (land or water) used to produce the biofuels. One must note that these predictions are based on 2002 farmland spatial distribution and fertilization rates, which were not expected to change by 2015 nor 2022. It is apparent from the results that shifting the U.S. energy supply from foreign oil to the farms of Midwest cannot occur without economic (land and crop) and environmental (water quality) impacts, which could potentially lead to more eutrophication in streams and hypoxia in the Gulf of Mexico. As a result, the ecosystems and the fishing industries would be adversely affected. Biofuels can be produced more sustainably by reducing the nitrogen flux resulting from crops production, hence reducing the adverse environmental impacts. Nitrogen management efforts could benefit from targeting nitrogen reductions in high impact stream catchments. However, the uncertainty from the model predictions could make it difficult to rank the priority of these catchments, especially at low flux catchments, since their predicted flux is very similar and the confidence interval in the predictions is around two-fold. Using riparian buffers could be an effective management practice to reduce nitrogen flux from anthropogenic inputs. Furthermore, given the price of cellulosic ethanol, farmers could profit from constructing switchgrass riparian buffers as a nitrogen management option. Growing switchgrass in riparian buffers could aid in meeting the 2022 mandates for cellulosic ethanol without competing for agricultural lands required to grow other crops or Conservation Reserve Program (CRP) lands that provide ecosystem services, such as reduced soil erosion, improved water quality and providing a wildlife habitat. In conclusion, meeting the biofuel mandates using any of the scenarios presented in this paper is not without its tradeoffs. The scope of this research only focused on nitrogen runoff and land requirements; however, further investigation requires assessing the economic costs and energy security implications of the biofuel mandates.

## Figures and Tables

**Table 1:** ESIA annual ethanol mandates (billion liters).

Year	Corn Ethanol	Cellulosic Ethanol	Other Sources, *i.e*., Biodiesel	Total
2011	48	1	4	53
2012	50	2	6	58
2013	52	4	7	63
2014	55	7	8	69
2015	57	11	9	78
2016	57	16	11	84
2017	57	21	13	91
2018	57	26	15	98
2019	57	32	17	106
2020	57	40	17	114
2021	57	51	17	125
2022	57	61	19	136

Source: [1].

**Table 2:** SPARROW calibration results of sources of nitrogen, delivery and decay variables.

Parameter	Units	Coefficient Units	NWLS Estimate	Confidence Interval		Standard Error	*p*-Value	Bootstrap Estimate
				Lower 90%	Upper 90%			
**Sources**								
Nitrogen applied to corn/soy	kg·N/year	Fraction	0.195	0.178	0.216	0.011	0.000	0.194
Nitrogen applied to hay	kg·N/year	Fraction	0.086	0.068	0.110	0.013	0.000	0.086
Nitrogen applied to other crops	kg·N/year	Fraction	0.006	−0.005	0.012	0.008	0.452	0.006
Atmospheric Deposition	kg·N/year	Fraction	0.110	0.057	0.159	0.030	0.000	0.110
Point discharge	kg·N/year	Fraction	0.727	0.534	0.912	0.142	0.000	0.729
Urban Land	km^2^	kg·N/km^2^/year	1210	863	1606	224	0.000	1207
Forests	km^2^	kg·N/km^2^/year	28.68	3.05	55.91	15.14	0.059	28.57
**Land-to-water delivery**								
Average Daily Temperature	Celsius	Celsius	−0.069	−0.087	−0.049	0.012	0.000	−0.069
Annual Total Precipitation	cm/year	cm/year	0.016	0.014	0.018	0.001	0.000	0.016
Wetlands	km^2^	km^2^	−0.007	−0.010	−0.003	0.002	0.003	−0.007
**Aquatic loss**								
Reach water time of travel	days	days^−1^	0.051	0.038	0.067	0.009	0.000	0.050
Reservoir residence time	days	days^−1^	0.002	0.002	0.003	0.001	0.000	0.002
**Summary Statistics**								
Number of sites		1003						
RMSE		0.57						
Adjusted *R*^2^		0.92						
Yield *R^2^*		0.87						
Shapiro–Wilk		0.92						
*p*-value		0.00						

**Table 3:** SPARROW calibration results of sources of nitrogen, delivery and decay variables.

Fuel	Mandate (Bil. Liters)		Conversion Factors (L/kg)	Yield *	Fertilization Rate (kg·N/m^2^)
	2015	2022			
Corn Ethanol	57	57	0.426 ^a^	3.58 kg/m^2 c^	0.015 ^d^
Cellulosic Ethanol	11	61	0.330 ^a^	0.5 kg/m^2^·(hay) ^c^ 0.9 kg/m^2^·(switchgrass) ^e^	0.0028 (hay) ^d^ 0.0112 (switchgrass) ^e^
Soybean Biodiesel	9	19	0.2–1.4 ^b^	1.10 kg/m^2^^c^	0.0027 ^d^

^a^ [14]; ^b^ [55,56]; ^c^ [57]; ^d^ [30]; ^e^ [58]; * yield = production/area planted for the year 2002.

**Table 4:** Average nitrogen flux, switchgrass production and potential cellulosic ethanol from high impact areas.

Year	Attribute	Total	Mean	SD
2015	Nitrogen Flux (MT)	191,319	481	182
	Catchment Area (km^2^)	179,136	450	295
	Stream Length (km)	17,266	43	19
	Potential Switchgrass (1000 MT)	15,325	-	-
	Cellulosic Ethanol (Billion liters)	5	-	-
2022	Nitrogen Flux (MT)	222,828	480	188
	Catchment Area (km^2^)	202,496	436	285
	Stream Length (km)	19,867	43	19
	Potential Switchgrass (1000 M.T)	17,849	-	-
	Cellulosic Ethanol (Billion liters)	5.9	-	-
